# Developing Pseudovirus-Based Neutralization Assay against Omicron-Included SARS-CoV-2 Variants

**DOI:** 10.3390/v14061332

**Published:** 2022-06-18

**Authors:** Hancong Sun, Jinghan Xu, Guanying Zhang, Jin Han, Meng Hao, Zhengshan Chen, Ting Fang, Xiangyang Chi, Changming Yu

**Affiliations:** Institute of Biotechnology, Academy of Military Medical Sciences, Beijing 100071, China; sun_hancong@163.com (H.S.); pjxjh333@163.com (J.X.); zhangguanying_123@outlook.com (G.Z.); harncy@126.com (J.H.); haorm_23@126.com (M.H.); czs0076@163.com (Z.C.); 15510742074@163.com (T.F.)

**Keywords:** SARS-CoV-2 variants, Omicron, pseudovirus, neutralization assay, convalescent plasma

## Abstract

The global spread of SARS-CoV-2 and its variants poses a serious threat to human health worldwide. Recently, the emergence of Omicron has presented a new challenge to the prevention and control of the COVID-19 pandemic. A convenient and reliable in vitro neutralization assay is an important method for validating the efficiency of antibodies, vaccines, and other potential drugs. Here, we established an effective assay based on a pseudovirus carrying a full-length spike (S) protein of SARS-CoV-2 variants in the HIV-1 backbone, with a luciferase reporter gene inserted into the non-replicate pseudovirus genome. The key parameters for packaging the pseudovirus were optimized, including the ratio of the S protein expression plasmids to the HIV backbone plasmids and the collection time for the Alpha, Beta, Gamma, Kappa, and Omicron pseudovirus particles. The pseudovirus neutralization assay was validated using several approved or developed monoclonal antibodies, underscoring that Omicron can escape some neutralizing antibodies, such as REGN10987 and REGN10933, while S309 and ADG-2 still function with reduced neutralization capability. The neutralizing capacity of convalescent plasma from COVID-19 convalescent patients in Wuhan was tested against these pseudoviruses, revealing the immune evasion of Omicron. Our work established a practical pseudovirus-based neutralization assay for SARS-CoV-2 variants, which can be conducted safely under biosafety level-2 (BSL-2) conditions, and this assay will be a promising tool for studying and characterizing vaccines and therapeutic candidates against Omicron-included SARS-CoV-2 variants.

## 1. Introduction

During the COVID-19 pandemic, the emergence of several highly transmissible SARS-CoV-2 variants, especially Omicron (B.1.1.529), has attracted enough attention worldwide. Some mutations in SARS-CoV-2 spike (S) protein can alter the antigenic properties with various distinct mechanisms, leading to their potential to be more transmissible, virulent, pathogenic, or evade immunity induced by previous infection or vaccination [1,2,3,4,5,6,7,8]. N501Y, which is present in lineages Alpha (B.1.17), Beta (B.1.351), Gamma (P.1), and Omicron, is responsible for higher affinity to ACE2 and increased infectivity due to a large phenolic group that makes two additional contacts with ACE2 residues [6,9,10]. Moreover, variants carrying E484K such as Beta and Gamma have been shown to contribute to the escape of some neutralizing antibodies and resistance to convalescent sera and postvaccination sera [10,11]. The replacement of a glutamate residue with a lysine residue causes a change in the biophysical properties of an epitope residue, diminishing some antibodies binding directly [12]. Moreover, lineages Beta and Gamma possess alternative amino acid substitutions K417N/T, which facilitate immune escape [13,14]. Regarding the Kappa (B.1.617.1) variant, the presence of the substitutions L452R and E484Q has been shown to affect antibody recognition, cause immune escape, and improve infectivity [15,16]. Currently, the Omicron variant contains a total of 59 mutations in its genome, with as many as 37 mutations occurring in the spike protein, including S371L, K417N, N440K, G446S, S477N, T478K, E484A, Q493R, G496S, Q498R, N501Y, and Y505H. In addition to some mutations that contribute to enhancing its transmissibility significantly [7,9,17], there are also some mutations that may affect partial therapeutic antibodies either through altering the conformation of mixed protein/carbohydrate epitope involving N343-N-linked glycan [18], such as S371L, or through changing its surface charge distribution, such as N440K, T478K, and E484A [7,19]. In Cao’s recent study, Omicron could lead to significant humoral immune evasion and potential antigenic shifting with more than 85% of the tested human-neutralizing antibodies being escaped [20].

Due to the high risk of SARS-CoV-2 infection, the cultivation of an authentic virus requires a laboratory with a high level of biosafety, at least a biosafety level 3 (BSL-3) laboratory equipped with a negative pressure system, which limits the throughput and accessibility of authentic virus neutralization assays. An alternative method is packaging convenient and reliable replication-defective pseudovirus expressing the S protein that can be used under BSL-2 conditions. To date, the development of SARS-CoV-2 pseudoviruses using human immunodeficiency virus (HIV)-based lentiviral particles [21,22], murine leukemia virus (MLV)-based retroviral particles [21,22,23], or vesicular stomatitis virus (VSV)-based systems [21,24,25,26] has become a powerful tool for evaluating the efficacy of therapeutic drugs and vaccines, and the results from such pseudovirus neutralization assays correlate well with the results of measurements using authentic viruses [22,27].

HIV-1 has been frequently utilized as a vector virus for the creation of pseudotyped viruses harboring foreign virus surface protein [28,29]. Its genome contains genes encoding three major viral structural proteins, namely, gag, pol, and env [30]; two regulatory proteins, namely, tat and rev; and four accessory proteins that help complete viral packaging, namely, vpr, vif, vpu, and nef [31]. In this study, we used an HIV-based lentiviral system to produce pseudoviruses displaying the spike protein of SARS-CoV-2 variants on their surface, in which the firefly luciferase gene was inserted into the pNL4-3 nef gene [32], leading to frame shifts in env and vpr. The SARS-CoV-2 pseudoviruses constructed from this system are replication-defective viruses, yet they are competent for a single round of infection to host cells in a similar way as authentic viruses. The pseudovirus-based neutralization assay generated in this work is safe, convenient, and useful for the evaluation of vaccines and therapeutic candidates against SARS-CoV-2 variants.

## 2. Materials and Methods

### 2.1. Plasmids and Cells

The full length of the S gene of Alpha (GISAID accession ID: EPI_ISL_708969), Beta (GISAID accession ID: EPI_ISL_712081), Gamma (GISAID accession ID: EPI_ISL_792680), Kappa (GISAID accession ID: EPI_ISL_1360306) or Omicron/BA.1 (GISAID accession ID: EPI_ISL_12422410) was codon-optimized, synthesized, and cloned into the pUC57 vector by General Biosystems Inc. (Anhui, China). The S genes of SARS-CoV-2 variants were then amplified using the primers 5′- TATCGATCCGGAGGTACCATGG-3′ and 5′- TTATCAGTGATGGTGATG-3′ and cloned into the expression vector pCAGGS with the NEBuilder^®^ HiFi DNA Assembly Cloning Kit (NEB), generating the pCAGGS-Alpha-S, pCAGGS-Beta-S, pCAGGS-Gamma-S, pCAGGS-Kappa-S, and pCAGGS-Omicron-S plasmids. The constructed recombinant plasmids bearing the S protein of SARS-CoV-2 variants were confirmed by DNA sequencing. The pDC316-WT-S containing a full-length S gene from Wuhan-Hu-1 and HIV backbone vector pNL4-3.Luc.R-E- were stored in our laboratory. ACE2-293T cells, which are HEK293T cells overexpressing ACE2 receptor, were produced and kept in our laboratory.

### 2.2. Analysis of SARS-CoV-2 Variant S Protein Expression

A total of 10^6^ HEK293T cells in 4 mL growth medium were seeded in each well of a 6-well plate 16 h before transfection. The pCAGGS-Alpha-S, pCAGGS-Beta-S, pCAGGS-Gamma-S, pCAGGS-Kappa-S, pCAGGS-Omicron-S, and the reference pDC316-WT-S plasmids were individually transfected into HEK293T cells. HEK293T cells transfected with an empty pCAGGS vector were used as the negative control. After 48 h of incubation, the cells were fixed with 100% methanol for 30 min at −20 °C and then blocked in PBS containing 2% FBS for 1 h at room temperature. The cells transfected with the pCAGGS-Omicron-S plasmid were then incubated with primary antibody (Sino Biological, 40591-MM41), which is specific to Omicron S protein, whereas the other cells were incubated with primary antibody (Sino Biological, 40591-MM43), which is specific to S proteins of SARS-CoV-2 variants except Omicron, at 5 µg/mL for 1 h at 37 °C, followed by a further incubation at 37 °C for 1 h with Alexa Fluor 488 goat anti-mouse secondary antibody (Abcam, ab150117) at 2 µg/mL. For nuclear staining, cells were treated with DAPI for 10 min at room temperature. Stained sections were analyzed with BioTek Cytation1 Cell Imaging Multi-Mode Plate Readers.

### 2.3. Production and Titration of SARS-CoV-2 Pseudotyped Variants

HEK293T cells were inoculated in cell dishes and grown overnight at 37 °C with 5% CO_2_ until the confluency for adherent cells reached 70–90%. The recombinant spike protein expression plasmids (pCAGGS-Alpha-S, pCAGGS-Beta-S, pCAGGS-Gamma-S, pCAGGS-Kappa-S, or pCAGGS-Omicron-S) were cotransfected with the HIV backbone vector pNL4-3.Luc.R-E- at different ratios into HEK293T cells with the Turbofect transfection reagent (Thermo Scientific, Waltham, MA, USA), respectively. The supernatants containing SARS-CoV-2 variant pseudoviruses were harvested 21–64 h after transfection and filtered through a 0.45 μm filter. The supernatants were then aliquoted into 2-mL cryotubes and stored at −80 °C.

The titer of the SARS-CoV-2 variant pseudovirus was measured by quantification of the luciferase activity. Supernatants containing SARS-CoV-2 variant pseudoviruses at the volume of 50 μL were used to infect 2 × 10^4^ ACE2-293T cells in 100 μL DMEM with 10% FBS in each well of 96-well plates. The well without the addition of the pseudovirus served as the cell control. After a 48-h incubation in a 5% CO_2_ environment at 37 °C, the culture supernatant was removed gently to leave 100 μL in each well, and then 100 μL of luciferase substrate (Perkin Elmer, Waltham, MA, USA) was added to each well. Two minutes after incubation at room temperature, 150 μL lysate was transferred to white solid 96-well plates (Costar, Washington, DC, USA) for the detection of luminescence using a TECAN Spark multifunctional microplate detector.

### 2.4. Neutralization Assay

For the neutralization assay, 50 μL pseudoviruses (~4 × 10^5^ RLU) were incubated with serial dilutions of plasma samples (dilutions of 1:10, 30, 90, 270, 810, 2430, 7290, and 21,870) from COVID-19 convalescent patients and healthy individuals or monoclonal antibodies for 1 h at 37 °C, and then 2 × 10^4^ ACE2-293T cells were added to each well. Cells without viruses, plasma, or antibodies were used as blank controls, and cells with viruses but without plasma or antibodies were used as virus controls. Luciferase activities were measured 48 h after infection, and the percent neutralization was calculated as 100% − (sample signals − blank control signals)/(virus control signals − blank control signals) × 100%. A three-parameter logistical analysis was performed on the full dilution series using Prism 8 (GraphPad Software, San Diego, CA, USA). All data are presented as the means ± standard deviations (SDs).

## 3. Results

### 3.1. Construction of the Recombinant Plasmids Expressing SARS-CoV-2 Variants Spike Proteins

**Figure 1:**
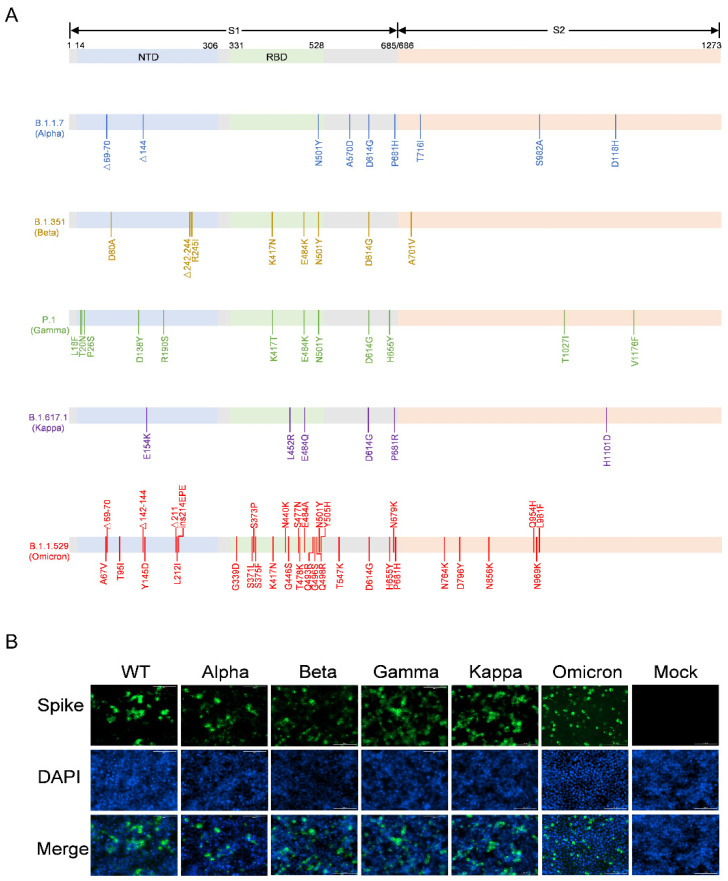
Detection of SARS-CoV-2 variants S protein expression in HEK293T cells. (**A**) Schematic overview of spike protein of SARS-CoV-2 variants, including Alpha (B.1.17), Beta (B.1.351), Gamma (P.1), Kappa (B.1.617.1), and Omicron (B.1.1.529). Amino acid mutations in comparison to the Wuhan-Hu-1 sequence are indicated. RBD, receptor binding domain; NTD, N-terminal domain. (**B**) Detection of SARS-CoV-2 S protein expression in HEK293T cells by immunofluorescence. The recombinant plasmids containing full-length S genes of SARS-CoV-2 variants were individually transfected into HEK293T cells. Cells transfected with an empty pCAGGS vector with the same procedure were used as the negative control. The cells were fixed after 48 h of incubation and labeled with the corresponding antibodies. Nuclei were stained with DAPI.

The full-length S protein genes of the Alpha, Beta, Gamma, Kappa, and Omicron variants (Figure 1A) were synthesized and individually cloned into the pCAGGS vector, generating the pCAGGS-Alpha-S, pCAGGS-Beta-S, pCAGGS-Gamma-S, pCAGGS-Kappa-S, and pCAGGS-Omicron-S recombinant plasmids, respectively. These recombinant plasmids and the pDC316-WT-S were transfected into HEK293T cells, and the expression of S proteins on the HEK293T cell surface was evaluated using immunofluorescence (Figure 1B). The results showed that the S proteins of the WT, Alpha, Beta, Gamma, Kappa, and Omicron variants were expressed on the surface of HEK293T cells, whereas HEK293T cells transfected with the empty pCAGGS vector did not express any S protein. These data suggest that the recombinant plasmids bearing the S protein of WT and its variants can be used to package pseudotyped viruses.

### 3.2. Optimization of SARS-CoV-2 Variants Pseudovirus Production

**Figure 2:**
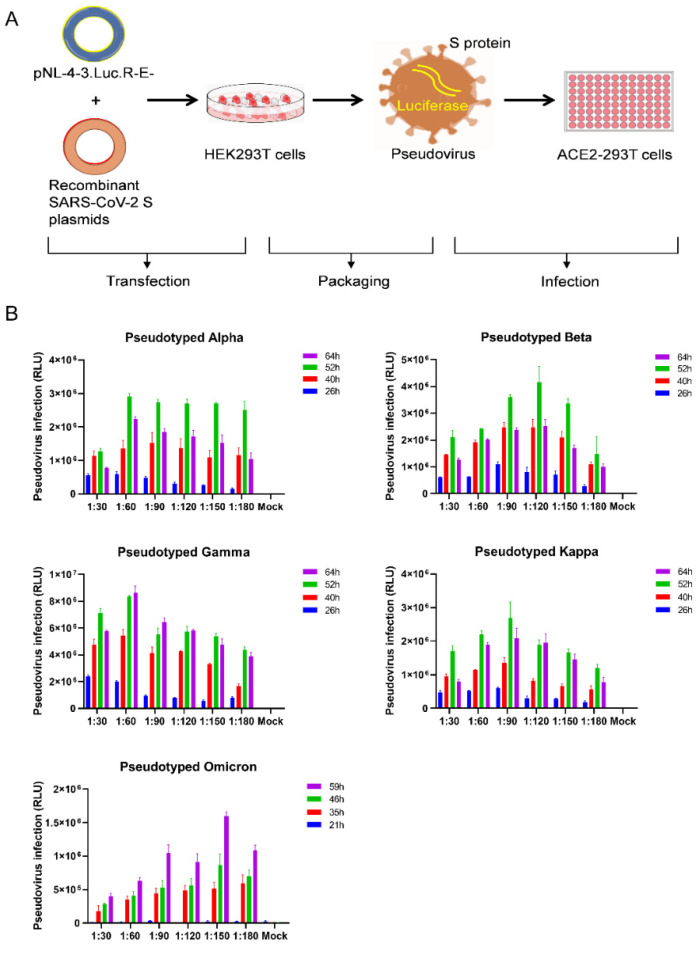
Optimization of SARS-CoV-2 variants pseudovirus production. (**A**) Schematic representation of the SARS-CoV-2 variants pseudovirus production and neutralization assay. The HIV backbone vector pNL4-3.Luc.R-E- plasmids were cotransfected with pCAGGS-Alpha-S, pCAGGS-Beta-S, pCAGGS-Gamma-S, pCAGGS-Kappa-S, or pCAGGS-Omicron-S, respectively into HEK293T cells to package the pseudotyped lentiviral particles. The supernatants containing SARS-CoV-2 variants pseudovirus with S protein were collected and then ACE2-293T cells were used to measure the pseudoviral titer. (**B**) Effect of the ratio of the recombinant S protein expression plasmids to the HIV backbone plasmids and the collection time for pseudovirus particles on the production of pseudovirus. Cells without pseudovirus infection were used as background. The data were expressed as mean relative luciferase units (RLU) ± standard deviation (SD) of 3 parallel wells in 96-well culture plates.

To generate the SARS-CoV-2 variant pseudoviruses, we used an HIV backbone vector-based pseudovirus packaging system (Figure 2A). The HIV backbone vector, pNL4-3.Luc.R-E-, was derived from pNL4-3 vector, in which the nef gene was replaced by the firefly luciferase gene, resulting in frame shifts in env and vpr. The recombinant spike protein expression plasmids (pCAGGS-Alpha-S, pCAGGS-Beta-S, pCAGGS-Gamma-S, pCAGGS-Kappa-S, or pCAGGS-Omicron-S) were cotransfected with the pNL4-3.Luc.R-E- plasmids into HEK293T cells, respectively, at ratios of 1:30, 1:60, 1:90, 1:120, 1:150, and 1:180. As shown in Figure 2B, the supernatants containing SARS-CoV-2 variant pseudoviruses were harvested 21–64 h post-transfection. ACE2-293T cells were infected with SARS-CoV-2 variant pseudoviruses for 48 h, and then viral titers were determined by measuring the relative luminescence units (RLU). The RLU values reached the peak level at 52 h post-infection for the pseudotyped Alpha, Beta, and Kappa variants at ratios of 1:60, 1:120, and 1:90, whereas the RLU level was highest for the Gamma variant at 64 h post-infection at a ratio of 1:60. For the Omicron pseudotyped virus, the highest viral titer was observed at 59 h after transfection at a ratio of 1:150 with approximately 1.6 × 10^6^ RLU.

### 3.3. Validation of the Neutralization Sensitivity of SARS-CoV-2 Pseudotyped Variants

**Figure 3:**
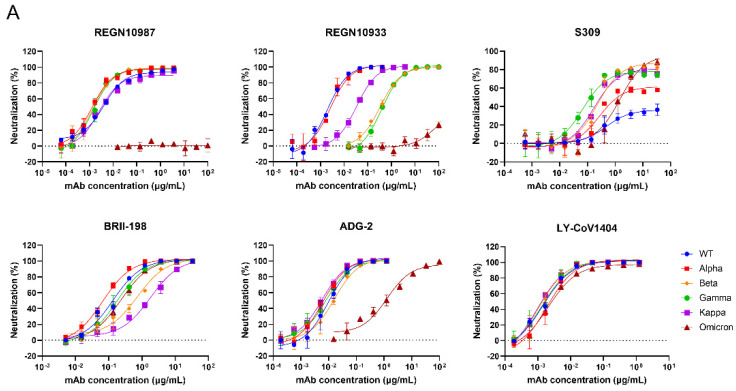

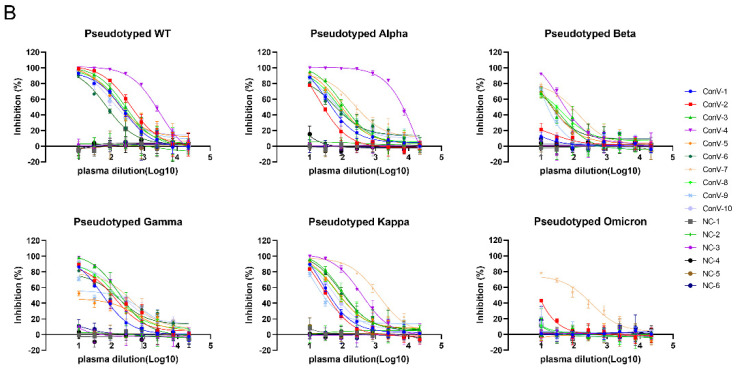
Validation of the neutralization sensitivity of SARS-CoV-2 pseudotyped variants. (**A**) Neutralizing curves of monoclonal antibodies against pseudotyped SARS-CoV-2 variants. Data are representative of at least two independent experiments. Mean ± SD was shown. (**B**) The inhibition activity of ten COVID-19 convalescent plasma samples against pseudotyped SARS-CoV-2 variants. Six plasma samples from healthy individuals were tested as negative controls (NC). The initial dilutions for both positive and negative samples were 1:10, followed by a 3-fold serial dilution. Samples were tested in triplicates and the experiments were repeated at least twice. Data from one of at least two independent experiments are presented in Mean ± SD.

To examine the neutralization sensitivity of the Omicron variant and the other SARS-CoV-2 variants, we evaluated the neutralizing activities of several antibodies that have obtained emergency use authorization (REGN10987 [33], REGN10933 [33], S309 [34], BRII-198 [35] and LY-CoV1404 [36]) or are being studied in clinical trials presently (ADG-2 [37]) against these SARS-CoV-2 pseudotyped variants (Figure 3A). Consistent with the previously reported results [20,38,39], Omicron/BA.1 can escape some neutralizing antibodies, such as REGN10987 and REGN10933, while S309 and ADG-2 function with reduced neutralization capability. Luckily, LY-CoV1404 and BRII-198 were able to potently neutralize Alpha, Beta, Gamma, Kappa, and Omicron/BA.1 pseudoviruses. Moreover, we assessed the neutralizing capacities of the plasma from ten COVID-19 convalescent patients [40], who recovered from infection of the Wuhan-Hu-1 strain, using the pseudovirus neutralization assays we established. As shown in Figure 3B, although all the plasma showed inhibition against pseudotyped wild-type (WT) virus, Alpha, Gamma, and Kappa variants with different average IC_50_ titers (462.9, 1818, 290.6, and 203.9), all of them we tested showed weak inhibition against Beta. When it turned to Omicron, only 1/10 (ConV-9) convalescent plasma showed detectable activity, and the reduction level was much higher than that of Beta. This result is consistent with the work of Cameroni et al. [41], indicating that Omicron was highly resistant to the plasma from the convalescent patients in Wuhan. Most control plasma samples from healthy individuals showed no inhibitory activity against all tested pseudoviruses, only few of them had extremely weak detectable responses, but the inhibition rates have not reached 50% in initial dilution.

## 4. Discussion

SARS-CoV-2 variants are highly pathogenic coronaviruses, and experiments with these viruses need to be performed in the biosafety level 3 laboratory with appropriate qualifications, which has become a bottleneck in drug and vaccine development. Pseudovirus neutralization assay based on retroviruses or HIV backbones is an alternative approach to authentic virus neutralization assay and can be used safely and conveniently to a greater extent for drug screening and vaccine evaluation [22,42,43,44].

Many factors affect the packaging efficiency of pseudoviruses, including the expression level of the viral envelope protein, the ratio of packaging plasmid combinations, the efficiency of plasmid transfection, the growth state of HEK293T cells before transfection, and the collection time of pseudovirus supernatants. In our study, the ratio of the spike protein expression plasmids to the HIV backbone plasmids considerably influenced the production of pseudovirus, and this ratio differed between wild-type SARS-CoV-2 and its variants. It is necessary to determine the optimal ratio of packaging plasmid combinations before large-scale pseudovirus packaging. Moreover, the optimal ratio of the spike protein expression plasmids to the HIV backbone plasmids in our study (1:60 to 1:120) were much lower than that in the previous studies (1:1 to 1:9) [21,45]. In addition, we found that a relatively high viral titer can be obtained from 50 h to 64 h after transfection, indicating that optimizing the collection time of pseudovirus supernatants is also crucial for improving the pseudovirus yield.

We used several approved monoclonal antibodies to validate the pseudovirus neutralization assay we established, including REGN10987, REGN10933, S309, BRII-198, LY-CoV1404, and ADG-2; the results were consistent with the previous research [20,38,39], which suggests this pseudovirus-based neutralization assay is a reliable alternative for the rapid detection of neutralizing antibodies against SARS-CoV-2 and its variants when BSL-3 facilities are not available. We also showed that convalescent plasma from COVID-19 patients blocked the entry of pseudotyped SARS-CoV-2 wild-type, Alpha, Beta, Gamma, and Kappa into ACE2-293T cells, while all convalescent plasma samples except ConV-9 failed to prevent the Omicron infection, underscoring the immune evasion of Omicron. However, one limitation of this assay was that we did not detect other receptors in addition to ACE2 receptor, the key determinant of SARS-CoV-2 attachment to target cells [46,47]. Recent studies have reported that SARS-CoV-2 also has AXL, KREMEN1, and ASGR1 in addition to ACE2 as its candidate receptors [48,49]. The role of other SARS-CoV-2 receptors in pseudovirus neutralization assay could not be ignored.

Overall, the spike proteins of SARS-CoV-2 variants were expressed on the surface of HEK293T cells to produce pseudovirus particles using a lentivirus vector-based pseudovirus system in optimized packaging conditions. This pseudovirus system can be used in animals to evaluate the in-vivo efficacy of vaccines or antibodies [50,51]. Taken together, this convenient and reliable pseudovirus system can be widely used for developing SARS-CoV-2 vaccines and therapeutic drugs and for studying SARS-CoV-2 infection.

## Data Availability

Not applicable.
